# Prevalence and incidence of atherosclerotic cardiovascular disease and its risk factors in Korea: a nationwide population-based study

**DOI:** 10.1186/s12889-019-7439-0

**Published:** 2019-08-14

**Authors:** Hyungtae Kim, Siin Kim, Sola Han, Pratik P. Rane, Kathleen M. Fox, Yi Qian, Hae Sun Suh

**Affiliations:** 1 College of Pharmacy, Pusan National University, Busandaehak-ro 63 beon-gil, Busan, South Korea; 2 Amgen, Inc, Thousand Oaks, CA USA; 3 Strategic Healthcare Solutions, LLC, Aiken, SC USA

**Keywords:** Atherosclerotic cardiovascular disease, Prevalence, Incidence

## Abstract

**Background:**

Atherosclerotic cardiovascular disease (ASCVD) is the leading cause of death in Korea. According to a report of published by Statistics Korea in 2014, cerebrovascular disease and cardiovascular disease were the major/leading causes of mortality. However, it is more difficult to identify prevalence and incidence of a disease than the mortality owing to the lack of national-level statistics. Few studies have examined the prevalence and incidence of ASCVD and its risk factors since 2012. This study aimed to estimate the prevalence and incidence of ASCVD and its risk factors in Korea using national claims data.

**Methods:**

We conducted a retrospective analysis using the national claims data of the Health Insurance Review and Assessment Service. Patients aged ≥18 years with ASCVD (defined as myocardial infarction, angina, coronary revascularization, peripheral artery disease, ischemic stroke, and transient ischemic attack) were identified between January 1, 2014 and December 31, 2015. Patients at high risk for ASCVD (defined as hypertension, diabetes mellitus, and dyslipidemia without ASCVD during the baseline period) were identified between January 1, 2015 and December 31, 2015. We estimated the prevalence, cumulative incidence, and incidence density. These were further stratified by age and sex. The respective denominators for prevalence and incidence were the census population and the at-risk population (defined as the population without respective disease 1 year prior to the respective disease identification).

**Results:**

Among the included Korean adult patients, the overall prevalence of clinical ASCVD per 1000 individuals was 98.25 in 2014 and 101.11 in 2015. The respective cumulative incidence and incidence density rates of ASCVD per 1000 individuals were 65.30 and 68.03 in 2014, and 67.05 and 69.94 in 2015, respectively. Peripheral artery disease seemed to drive the increase in the total prevalence and incidence of ASCVD. The prevalence and incidence of ASCVD continued to increase with age until 79 years.

**Conclusions:**

This national population-based study confirmed the high prevalence and incidence of ASCVD and its risk factors in the adult population of South Korea. We suggest that more intensive treatment and prevention are needed to prevent ASCVD.

**Electronic supplementary material:**

The online version of this article (10.1186/s12889-019-7439-0) contains supplementary material, which is available to authorized users.

## Background

Atherosclerotic cardiovascular disease (ASCVD) is one of the main causes of death worldwide. In 2015, up to 31% of global deaths were due to ASCVD [1]. Within the USA and EU, ASCVD accounts for 33–40% of all-cause mortality at any age and a total economic toll of $297.7 billion and €196 billion in 2008, respectively. ASCVD is the primary cause of death in the EU, and the burden of diseases from ASCVD in the USA is greater than that from any other chronic diseases [2]. In South Korea, cardiovascular disease, including cardiac disease (i.e., myocardial infarction, angina, and heart failure) and cerebrovascular disease are the major/leading causes of death. Cardiovascular disease accounts for 1 in every 5 deaths [3].

Korea has made extensive effort to manage ASCVD through establishment of comprehensive cardiac and cerebrovascular disease care centers, and development of guidelines for diseases related to ASCVD [4–7]. In recent years, the Korean Heart Study cohort was established to improve the understanding of risk factors for ASCVD and to study disease management with government support [8]. Despite these efforts, studies on the prevalence and incidence of ASCVD and its risk factors in the entire population are lacking, unlike studies on ASCVD mortality. Epidemiology studies on prevalence and incidence can provide information on disease frequency, support to identify the burden of disease, and help in establishing a treatment strategy [9]. Moreover, an assessment of the epidemiology of ASCVD and its risk factors in small population may be limiting the understanding of the immense impact on overall population and societal health. Therefore, we conducted an epidemiological study to determine the recent prevalence and incidence of ASCVD and its risk factors in entire population.

## Methods

### Data source

This study used the most recent data available from the Health Insurance Review and Assessment Service (HIRA) database, which included claims data sourced from the entire population of South Korea between January 1, 2013 and December 31, 2015. The HIRA is a government agency that oversees and evaluates healthcare insurance expenses covering the entire population of about 50 million (the Korean National Health Insurance covers approximately 97%, and Medical Aid covers approximately 3%) in Korea [10]. The claims data of the HIRA contains information on patient diagnoses, treatments, procedures, surgical histories, and prescription drugs across the full range of healthcare settings regardless of geographic location [11].

### Study population

Patients aged over 18 years with ASCVD were selected from January 1, 2014 to December 31, 2015, and those at high risk for ASCVD were selected from January 1, 2015 to December 31, 2015. The intake period of patients at high risk for ASCVD was set to 1 year. All patients with at least 1 diagnosis, medication, or procedure code related to ASCVD, or a risk factor for ASCVD were identified. Additional file 1: Table S1 shows the Korean Classification of Disease, 6th Revision (KCD-6) codes used to define these conditions, and reflects the domestic health and medical care environment in Korea according to the International Classification of Diseases, 10th Revision diagnosis codes [12]. The codes and definitions of medications and procedures are described in Additional file 2: Table S2 and Additional file 3: Table S3.

**Fig. 1:**
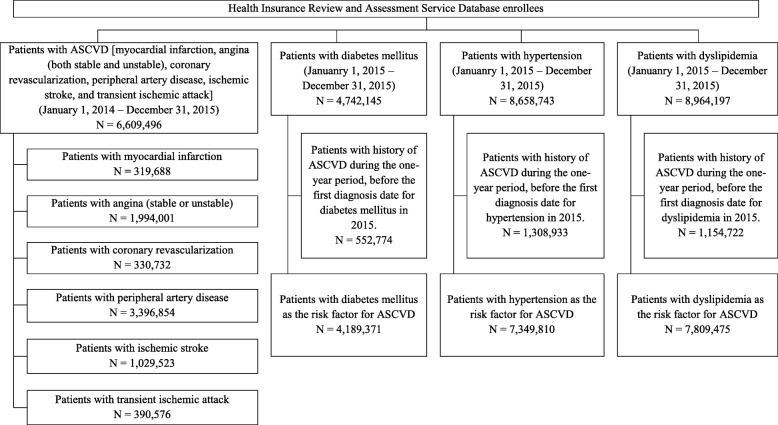
Selection of the study participants from the Health Insurance Review and Assessment Service Database. ASCVD, atherosclerotic cardiovascular disease

A sample selection flow chart including the intake period is presented in Fig. 1. Patients with ASCVD were defined as those with myocardial infarction, angina (stable or unstable), coronary revascularization, peripheral artery disease, ischemic stroke, or transient ischemic attack (TIA), according to the American College of Cardiology and American Heart Association (ACC/AHA) 2013 guidelines [13]. Patients at high risk for ASCVD were defined as those with diabetes mellitus, hypertension, or dyslipidemia without a history of ASCVD according to the Adult Treatment Panel (ATP) III 2001 guidelines and Korean dyslipidemia treatment guidelines [14, 15]. The index date was defined as that of the first disease diagnosed in each year. When patients had multiple diseases of interest as the first diagnosis, the data for each were used to estimate the incidence and prevalence for each disease of interest. Patient demographics, including sex and age, were reported on the index date. The pre-index period was defined as the 12-month period before the index date and was used to characterize patient characteristics to determine whether the disease on the index date was a new case. The pre-index period was also used to determine whether any ASCVD existed before the risk factors for ASCVD occurred. The incident patients included those without a history of ASCVD or risk factors for ASCVD during the pre-index period. For example, if there was at least 1 diagnosis or treatment related to ASCVD 1 year before the index date of hypertension, then hypertension was not considered as a risk factor for ASCVD, and this case was excluded in the groups of patients with a high risk for ASCVD.

### Outcome measures

The prevalence, cumulative incidence, and incidence density were reported for each year by disease. All results were stratified by 10-year age groups and sex. The annual prevalence was estimated as the number of patients (aged ≥18 years) with the disease divided by the census population (aged ≥18 years). The annual cumulative incidence was measured as the number of incident patients divided by the number of population at risk (aged ≥18 years) which was calculated from the number of census population minus the number of prevalence patients in the previous year. The annual incidence density was estimated as the number of incident patients divided by the number of person-years accumulated in the population without any ASCVD in all groups or without each risk factor for ASCVD in the respective groups. The person-years were calculated as: [total census population of the year − number of pre-existing cases in the previous year – Σ (days from occurrence of case to the end of the year)*/365] (*Only patients without preexisting disease in the previous year were followed up.). The prevalence and incidence estimates were adjusted for the standard population of Korea in 2005 according to age.

### Statistical analyses

The mean values, medians, ranges, and standard deviations were estimated for continuous variables, and the frequencies and proportions were calculated for categorical variables. Statistical analyses were conducted using the SAS (version 9.3) software program (SAS Institute, Inc., Cary, NC, USA).

## Results

### Demographics

The number of patients with ASCVD was 4,073,832 in 2014 and 4,235,437 in 2015. Approximately 60% of these patients were incident patients in both years. The number of patients with disease was slightly higher in women than in men. When we excluded the patients with ASCVD in the previous year in these groups, the numbers of patients at high risk for ASCVD in 2015 were estimated as follows: 4,189,371 (diabetes mellitus), 7,349,810 (hypertension), and 7,809,475 (dyslipidemia) (Table 1 and Additional file 4: Table S4).

**Table 1:** Baseline characteristics of the patients with ASCVD and at high risks for ASCVD

Characteristics	2014				2015			
	Patients to estimate prevalence		Patients to estimate incidence		Patients to estimate prevalence		Patients to estimate incidence	
Patients with ASCVD								
Number of patients	4,073,832		2,431,291		4,235,437		2,535,664	
Age at index (mean, SD)	63.77	13.40	62.04	14.20	64.07	13.43	62.31	14.20
Male (n, %)	1,917,135	47.1%	1,115,023	45.9%	2,004,384	47.3%	1,171,480	46.2%
Patients with diabetes					4,742,145			
Patients with diabetes without ASCVD								
Number of patients					4,189,371		1,368,326	
Age at index (mean, SD)					60.87	13.38	57.22	14.68
Male (n, %)					2,158,643	51.5%	673,252	49.2%
Patients with hypertension					8,658,743			
Patients with hypertension without ASCVD								
Number of patients					7,349,810		1,052,252	
Age at index (mean, SD)					62.50	12.97	56.16	14.34
Male (n, %)					3,605,515	49.1%	581,354	55.3%
Patients with dyslipidemia					8,964,197			
Patients with dyslipidemia without ASCVD								
Number of patients					7,809,475		3,265,877	
Age at index (mean, SD)					57.21	14.11	52.45	15.16
Male (n, %)					3,561,808	45.6%	1,522,747	46.6%

*ASCVD* atherosclerotic cardiovascular disease, *CCI* Charlson Comorbidity Index, *SD* standard deviation, *HIV* human immunodeficiency virus, *AIDS* acquired immunodeficiency syndrome

### Prevalence and incidence of ASCVD

**Fig. 2:**
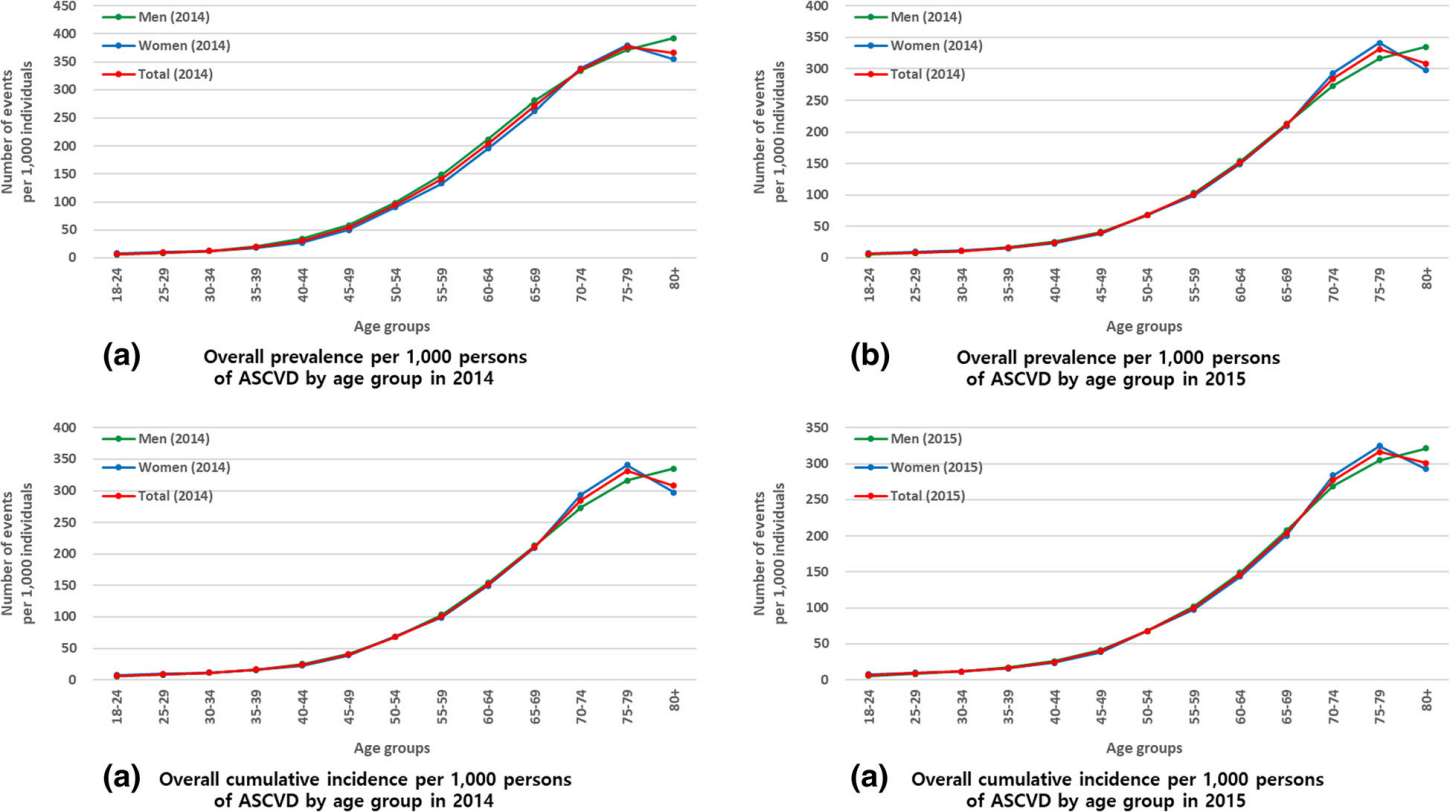
Prevalence and cumulative incidence of ASCVD by age group in 2014 and 2015. ASCVD, atherosclerotic cardiovascular disease

**Fig. 3:**
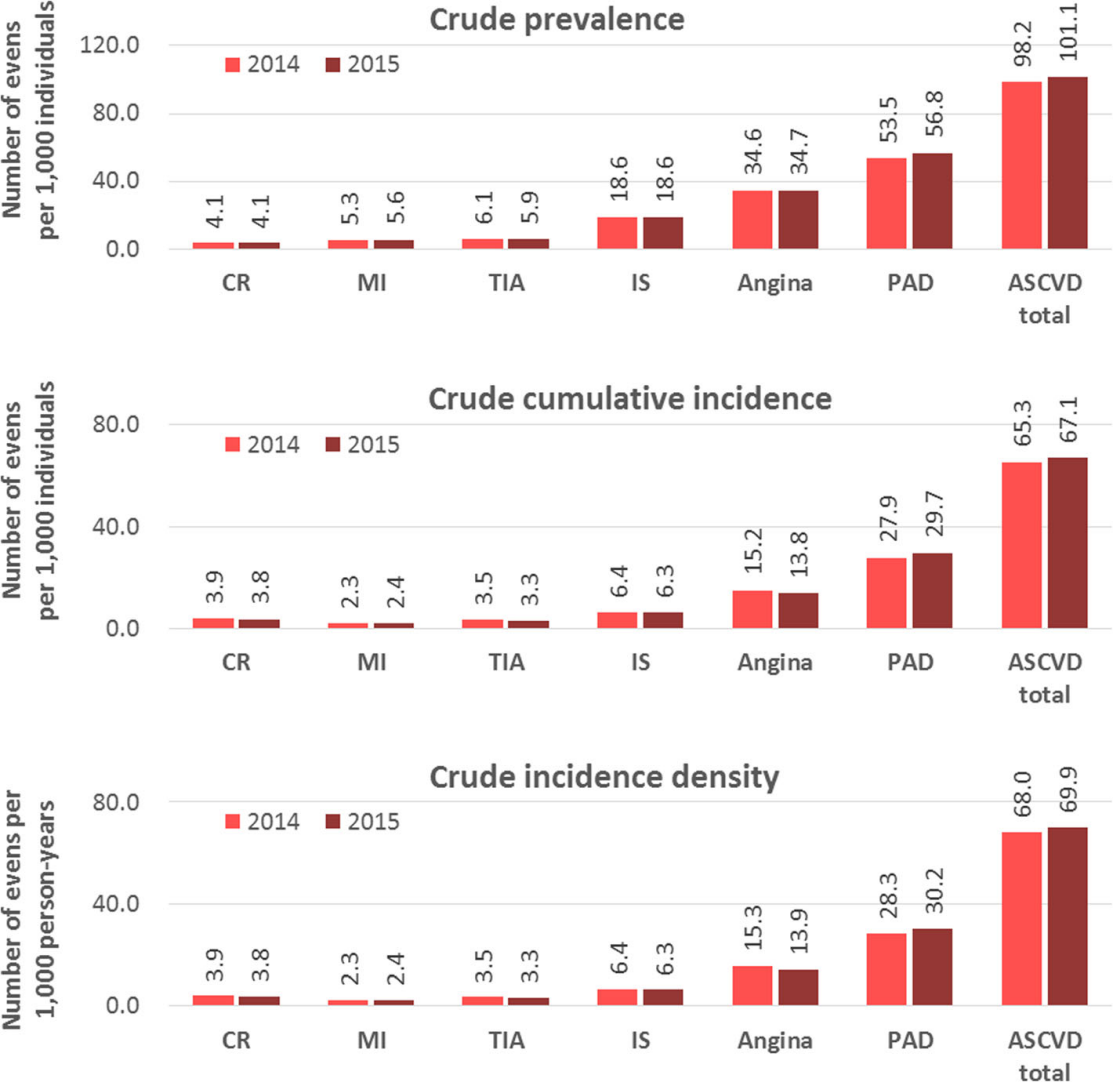
Prevalence, cumulative incidence, and incidence density of ASCVD by year. ASCVD, atherosclerotic cardiovascular disease; MI, myocardial infarction; CR, coronary revascularization; PAD, peripheral artery disease; IS, ischemic stroke; TIA, transient ischemic attack

The crude total prevalence of ASCVD per 1000 individuals was 98.25 in 2014 and 101.11 in 2015. The crude cumulative total incidence of ASCVD per 1000 individuals was 65.30 in 2014 and 67.05 in 2015. The age-adjusted prevalence and cumulative incidence of ASCVD per 1000 individuals were 77.81 and 61.47 in 2014 and 78.07 and 60.32 in 2015, respectively (Table 2). The incidence density of ASCVD per 1000 person-years was 68.03 in 2014 and 69.94 in 2015. The prevalence and cumulative incidence of total ASCVD increased with age and peaked in the 70–79-year age group (Fig. 2 and Additional file 5: Table S5). The patients with the highest crude prevalence and cumulative incidence were those with peripheral artery disease (PAD) (per 1000 individuals: 53.49 in 2014 and 56.75 in 2015), followed by angina (per 1000 individuals: 34.59 in 2014 and 34.69 in 2015) and ischemic stroke (per 1000 individuals: 18.69 in 2014 and 18.62 in 2015) (Fig. 3 and Additional file 5: Table S5).

**Table 2:** Prevalence, cumulative incidence, and incidence density of ASCVD and risk factors for ASCVD

Disease	2014									2015								
	Prevalence (Rate per 1000 individuals)			Cumulative incidence (Rate per 1000 individuals)			Incidence density (Rate per 1000 PYs)			Prevalence (Rate per 1000 individuals)			Cumulative incidence (Rate per 1000 individuals)			Incidence density (Rate per 1000 Pys)		
	Male	Female	Total	Male	Female	Total	Male	Female	Total	Male	Female	Total	Male	Female	Total	Male	Female	Total
ASCVD Total																		
Crude population	93.27	103.14	98.25	60.07	70.51	65.30	62.40	73.65	68.03	96.52	105.62	101.11	62.15	71.93	67.05	64.66	75.22	69.94
Age-adjusted^a^	72.48	82.96	77.81	54.97	67.94	61.47	61.65	77.79	69.71	72.95	82.97	78.07	54.29	66.29	60.32	60.62	75.46	68.06
Angina																		
Crude population	36.10	33.10	34.59	15.18	15.20	15.19	15.30	15.32	15.31	36.65	32.77	34.69	14.07	13.61	13.84	14.17	13.71	13.93
Age-adjusted^a^	28.05	26.38	27.29	12.86	13.03	12.95	13.05	13.24	13.14	27.53	25.32	26.52	11.61	11.37	11.50	11.76	11.53	11.65
Ischemic stroke																		
Crude population	18.66	18.55	18.60	6.15	6.70	6.43	6.17	6.72	6.45	18.83	18.41	18.62	6.04	6.51	6.28	6.06	6.53	6.30
Age-adjusted^a^	13.53	13.93	13.81	4.74	5.37	5.07	4.79	5.43	5.13	13.15	13.38	13.35	4.46	5.02	4.76	4.50	5.08	4.81
Myocardial infarction																		
Crude population	7.12	3.60	5.34	2.72	1.90	2.31	2.72	1.90	2.31	7.54	3.70	5.61	2.79	1.93	2.36	2.79	1.93	2.36
Age-adjusted^a^	5.48	2.75	4.16	2.16	1.50	1.84	2.16	1.50	1.84	5.64	2.75	4.25	2.15	1.49	1.83	2.16	1.50	1.84
Transient ischemic attack																		
Crude population	5.44	6.81	6.13	3.06	3.92	3.49	3.06	3.93	3.50	5.25	6.46	5.86	2.91	3.64	3.28	2.91	3.65	3.28
Age-adjusted^a^	4.16	5.43	4.80	2.42	3.22	2.82	2.43	3.23	2.83	3.91	5.03	4.47	2.25	2.93	2.59	2.25	2.94	2.60
Coronary revascularization																		
Crude population	5.12	3.14	4.12	4.74	2.98	3.85	4.75	2.99	3.86	5.13	3.05	4.08	4.78	2.91	3.83	4.79	2.91	3.84
Age-adjusted^a^	3.98	2.47	3.25	3.70	2.36	3.05	3.72	2.37	3.06	3.87	2.33	3.13	3.63	2.24	2.95	3.65	2.25	2.97
Peripheral artery disease																		
Crude population	46.89	59.97	53.49	23.19	32.62	27.91	23.46	33.16	28.30	49.97	63.42	56.75	24.93	34.49	29.72	25.24	35.08	30.16
Age-adjusted^a^	36.56	48.86	42.70	19.68	29.04	24.32	20.15	29.98	25.01	38.02	50.64	44.32	20.63	30.00	25.27	21.13	30.95	25.99
Risk factors for ASCVD																		
Diabetes																		
Crude population										103.95	96.14	100.01	36.94	37.08	37.01	37.63	37.77	37.70
Age-adjusted^a^										82.92	77.80	80.36	33.74	34.74	34.19	34.77	35.93	35.29
Hypertension																		
Crude population										173.63	177.26	175.46	34.96	28.15	31.54	35.58	28.55	32.04
Age-adjusted^a^										136.81	138.15	137.29	35.23	32.87	33.75	36.38	34.35	35.02
Dyslipidemia																		
Crude population										171.52	201.09	186.43	91.08	106.33	98.63	95.38	112.24	103.70
Age-adjusted^a^										146.06	169.94	157.64	88.48	109.44	98.24	94.20	119.64	105.86

*ASCVD* atherosclerotic cardiovascular disease, *Pys* person-years

^a^These morbidities are annually age adjusted for the standard population of South Korea in 2005

### Prevalence and incidence of the risk factors for ASCVD

**Fig. 4:**
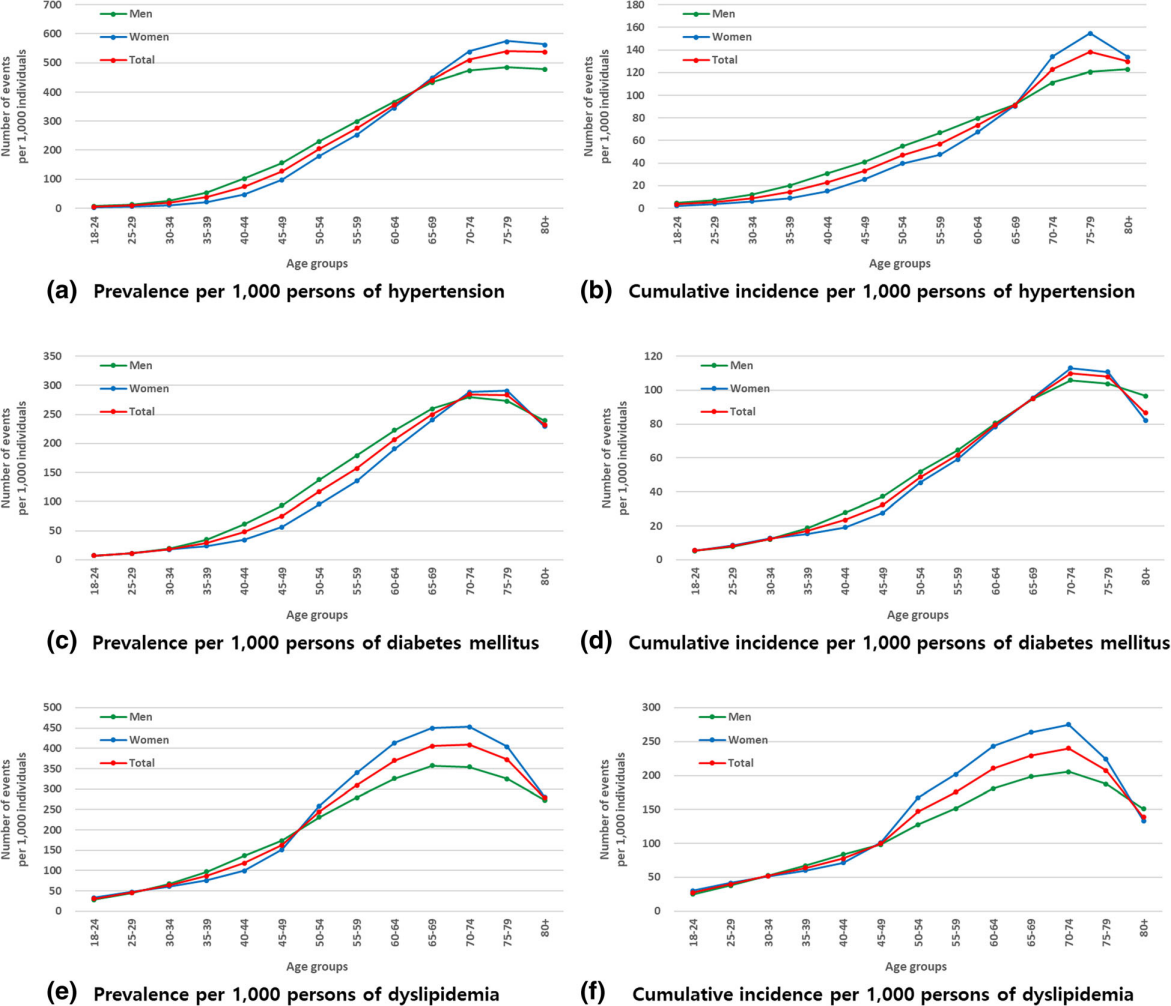
Overall prevalence and cumulative incidence of the risk factors for ASCVD by age group. ASCVD, atherosclerotic cardiovascular disease

Dyslipidemia had the highest prevalence and cumulative incidence among the risk factors for ASCVD. The prevalence of diabetes mellitus, hypertension, and dyslipidemia was 100.01, 175.46, and 186.43 per 1000 individuals in 2015, respectively; their cumulative incidence was 37.01, 31.54, and 98.63 in the same year, respectively (Table 2). The groups with the highest crude prevalence and cumulative incidence were somewhat different by each risk factor for ASCVD, age, and sex (Fig. 4 and Additional file 6: Table S6). All values and tendency of increasing and decreasing incidence densities were similar to those of cumulative incidence (Fig. 3 and Table 2).

## Discussion

This population-based study was a large-scale retrospective analysis conducted to explore the recent prevalence and incidence of ASCVD and its risk factors, including diabetes mellitus, hypertension, and dyslipidemia, using national claims data. The total prevalence and cumulative incidence of ASCVD slightly increased in 2015, compared to 2014. The main disease that increased the prevalence and incidence was PAD. The prevalence and incidence increased with age regardless of ASCVD disease types. This study is important since the epidemiology of all ASCVD and its risk factors were examined in a nationwide population. Thus, the estimates for several diseases shown in this study are comparable to each other and could be used as basic epidemiological information in other studies.

Previous study reported that the prevalence and cumulative incidence of cardiovascular disease (KCD-6 codes: I20, I21, I22, I23, I24, I25, I50, I70, and I71) were 6.76 and 1.84 per 100 individuals, respectively. The previous report also estimated the prevalence and cumulative incidence of diabetes mellitus (13.19 and 3.55 per 100 individuals, respectively), hypertension (26.30 and 3.37 per 100 individuals, respectively), and dyslipidemia (19.60 and 6.94 per 100 individuals, respectively) [16]. The prevalence and incidence were similar but slightly different from our results because the study period and codes used to define the disease were slightly different. However, our estimate of total ASCVD prevalence was generally consistent with that of other countries. According to a recent European epidemiological study of cardiovascular disease, the percentage of patients with cardiovascular disease by country ranged from 4.3 to 17.7% in 2014. The results of this study also showed a similar prevalence of cardiovascular disease between men and women in Europe [17]. The total prevalence of ASCVD in our study was between 98.25 and 101.11 per 1000 individuals and these results were within the range of prevalence in Europe. Another study reported that the prevalence of ASCVD or diabetes was about 15% in USA [18]. The prevalence of ASCVD and diabetes without ASCVD in our study was 101.11 and 100.01 per 1000 individuals, respectively.

Our study demonstrated that the overall prevalence and cumulative incidence of ASCVD slightly increased in 2015 compared with those in 2014, and the most prevalent types of ASCVD were PAD, angina, and ischemic stroke. The prevalence and cumulative incidence of most types of ASCVD showed a slightly decreasing or similar trend in 2015 compared with those in 2014. However, the incidence of PAD increased compared with those of other types of ASCVD. This seemed to drive an increase in the total prevalence and incidence of ASCVD. PAD is an important part of CVD and strongly associated with ASCVD mortality [19–26]. Moreover, PAD has itself been described as a vascular disease and risk factor for ASCVD at the same time [27]. Previous studies showed that 1–15% have cardiovascular disease among those with PAD [22, 28]. In our study, patients with ASCVD tended to have comorbid PAD (among the prevalence group, 32.1% in 2014 and 33.0% in 2015) compared with other cardiovascular diseases including myocardial infarction (4.3% in 2014 and 4.4% in 2015), congestive heart failure (10.1% in 2014 and 10.8% in 2015), and cerebrovascular disease (25.7% in 2014 and 25.6% in 2015). Similarly, in a previous study that estimated the prevalence of PAD, patients with other vascular diseases (coronary artery disease or cerebrovascular disease) had a significantly higher prevalence than the control group with no coronary artery disease or cerebrovascular disease in Korea [29]. Based on these results, further investigation on the relationship between PAD and other types of ASCVD might be needed.

The prevalence and incidence of ASCVD tended to increase with age regardless of the type of disease. These trends were similar to those in previous studies [16, 30, 31]. However, the prevalence and incidence in men and women varied by disease, although the total prevalence and incidence of ASCVD for patients aged over 18 years were higher in women than in men. Transient ischemic attack and PAD had a high prevalence and incidence in women. Compared to those reported in the USA, sex-specific trends were consistent for some diseases, but not for others. For example, the recent statistics from the AHA reported that the prevalence of angina and myocardial infarction were higher in men than those in women, and this tendency can be seen in our study. However, the incidence of transient ischemic attack was higher in men than in women, and this is contrary to our findings [32]. Additionally, the prevalence and cumulative incidence of risk factors in our study showed slightly different trends in terms of age and sex by disease (diabetes, hypertension, and dyslipidemia). For example, the age group with the highest prevalence for each disease was 70–74 for diabetes, 75–79 for hypertension, and 65–69 for dyslipidemia. The highest cumulative incidence by age group was 70–74 for diabetes, 75–79 for hypertension, and 70–74 for dyslipidemia. Prevalence was higher in females than in males in those aged 18–29 and 70–79 years of age for diabetes, 65 and older for hypertension, and 18–29 and 50 years of age in for dyslipidemia. These diseases are well-known risk factors for ASCVD as identified in multiple guidelines and previous studies [5, 33, 34]. The results in our study suggest that it would be better to set up management strategies to prevent ASCVD tailored to each patient according to the age, sex, and history of disease.

Our study has several distinct strengths. First, we conducted this study using the national claims data from a universal health coverage system that covers the entire population of South Korea [11]. Therefore, our findings are representative of the entire South Korean population. Additionally, the results of this study were estimated for each type of ASCVD and the risk factors for ASCVD, and stratified by age and sex; thus, the data are considerably useful as scientific evidence.

Despite these strengths, the limitations of a retrospective study using insurance data may be present, because claims data are primarily used for financial and administrative management rather than research [35]. However, since the claims data that we used covered the entire national population, prevalence and incidence results of this study can be generalized to the Korean population. Additionally, code accuracy and validity can be an important issue. First, this study used diagnosis, procedure, and medication codes to identify the diseases recorded for reimbursement of healthcare services, making it susceptible to coding errors. However, a previous validation study reported that the agreement rate between diagnosis codes in HIRA data and diagnoses in chart review was about 70% [36]. The accuracy of the codes improved for cardiovascular disease or according to increasing disease severity [36–38]. The validity of diagnosis codes associated with diabetes has also been verified [39]. Moreover, codes used in this study were based on preceding studies and carefully reviewed by several experts including clinicians [40–44]. Second, we identified diseases using only diagnosis codes, except for dyslipidemia. Because several medications have various indications, we did not use medication codes to identify diseases to avoid misclassification bias. Third, the prevalence and incidence might be underestimated because undiagnosed patients cannot be identified using claims data. Finally, we estimated the incidence rates using a 1-year of disease-free period as a commonly used baseline in claims database analysis. This relatively shorter disease-free period might have resulted in relatively higher incidence rates [45]. Thus, caution is needed when interpreting the incidence results. Other issues related to unmeasured confounders in this retrospective study using claims data were not a problem because our study did not use a comparison group.

From the perspective of health services research, the latest status of ASCVD determined by this study using the claims data covering the entire national population can be used as an important basis for estimating the burden of ASCVD. This study can also support the decision-making process related to ASCVD in Korea. The prevalence and incidence estimated though epidemiological study can help to establish appropriate strategies for managing disease [46]. For example, when conducting quantitative cost-effectiveness analysis using a decision-making model, transition probabilities can be estimated using a variety of data sources including epidemiological studies or administrative data [47]. Other types of decision-making research including cost-of-illness studies to promote attention to public policy and stimulate debate also require information on prevalence and incidence to estimate the burden of disease [48].

## Conclusions

This population-based study explored the recent prevalence and incidence of ASCVD and its risk factors, including diabetes mellitus, hypertension, and dyslipidemia. High prevalence and incidence of ASCVD and its risk factors in the adult population in Korea were observed, with a marked increase in PAD. We suggest that more intensive management strategies are needed to reduce the burden of ASCVD.

## Additional files

Additional file 1: **Table S1.** Diseases, risk factors, and codes for patients with ASCVD or at a high risk for ASCVD (XLSX 11 kb)

Additional file 2: **Table S2.** Procedure codes and names for patients with ASCVD (XLSX 13 kb)

Additional file 3: **Table S3.** Medication codes to identify dyslipidemia (XLSX 11 kb)

Additional file 4: **Table S4.** Baseline characteristics of the patients with ASCVD and at high risks for ASCVD (XLSX 18 kb)

Additional file 5: **Table S5.** Prevalence, cumulative incidence, and incidence density of ASCVD in 2014 and 2015 (XLSX 39 kb)

Additional file 6: **Table S6.** Prevalence, cumulative incidence, and incidence density of the risk factors (diabetes, hypertension, and dyslipidemia) for atherosclerotic cardiovascular disease in 2015 (XLSX 17 kb)

## Data Availability

The claims data provided by the Health Insurance Review and Assessment Service (M20160519311, M20160926461, M20160927465, M20160927467, M20160921456) and analyzed during this study are not publicly available according to HIRA data protection regulations. Thus, we cannot share the data we used for this study with other researchers.

## Abbreviations

ACC/AHA: American College of Cardiology and American Heart Association
AHA: American Heart Association
ASCVD: Atherosclerotic cardiovascular disease;
ATP: Adult Treatment Panel
CR: Coronary revascularization
EU: European Union
HIRA: Health Insurance Review and Assessment
IS: Ischemic stroke
KCD: Korean Classification of Disease
MI: Myocardial infarction
PAD: Peripheral artery disease
TIA: Transient ischemic attack
US: United States
